# No Association Between *GRM3* and Japanese Methamphetamine-Induced Psychosis

**DOI:** 10.2174/157015911795017001

**Published:** 2011-03

**Authors:** Tomoko Tsunoka, Taro Kishi, Masashi Ikeda, Tsuyoshi Kitajima, Yoshio Yamanouchi, Yoko Kinoshita, Kunihiro Kawashima, Tomo Okochi, Takenori Okumura, Toshiya Inada, Hiroshi Ujike, Mitsuhiko Yamada, Naohisa Uchimura, Ichiro Sora, Masaomi Iyo, Norio Ozaki, Nakao Iwata

**Affiliations:** 1 Department of Psychiatry, Fujita Health University School of Medicine, Toyoake, Aichi 470-1192, Japan; 2 Japanese Genetics Initiative for Drug Abuse, Japan; 3 Department of Psychiatry, Seiwa Hospital, Institute of Neuropsychiatry, Tokyo 162-0851, Japan; 4 Department of Neuropsychiatry, Okayama University Graduate School of Medicine, Dentistry and Pharmaceutical Sciences, Okayama 700-8558, Japan; 5 National Institute of Mental Health, National Center of Neurology and Psychiatry, Ichikawa 272-0827, Japan; 6 Department of Neuropsychiatry, Kurume University School of Medicine, Kurume 830-0011, Japan; 7 Department of Psychobiology, Department of Neuroscience, Tohoku University Graduate School of Medicine, Sendai 980-8576, Japan; 8 Department of Psychiatry, Chiba University Graduate School of Medicine, Chiba 260-8677, Japan; 9 Department of Psychiatry, Nagoya University Graduate School of Medicine, Nagoya 466-8850, Japan; 10 Department of Psychological Medicine, School of Medicine, Cardiff University, Heath Park, Cardiff, CF14 4XN, United Kingdom

**Keywords:** GRM3, Methamphetamine-Induced Psychosis, case-control study.

## Abstract

Several investigations have suggested that abnormalities in glutamate neural transmission play a role in the pathophysiology of psychiatric disorders, including schizophrenia. The metabotropic glutamate 3 receptor (mGluR3) gene was reported to be associated with schizophrenia, and paranoid type schizophrenia has symptoms that are similar to those of methamphetamine-induced psychosis. This suggests that mGluR3 gene (*GRM3*) is a good candidate gene for the pathogenesis of methamphetamine-induced psychosis. To evaluate the association between *GRM3* and methamphetamine-induced psychosis, we conducted a case-control study of Japanese samples (181 methamphetamine-induced psychosis and 232 controls).

**Methods:**

We selected one functional SNP (rs6465084), reported to be associated with prefrontal brain functioning, for an association analysis. Written informed consent was obtained from each subject. This study was approved by the ethics committees at Fujita Health University, Nagoya University Graduate School of Medicine and each participating member of the Institute of the Japanese Genetics Initiative for Drug Abuse (JGIDA).

**Results:**

We did not detect an association between rs6465084 in *GRM3* and Japanese methamphetamine-induced psychosis.

**Conclusion:**

Our findings suggest that rs6465084 in *GRM3* does not play a major role in the pathophysiology of methamphetamine-induced psychosis in the Japanese population. However, because we did not perform an association analysis based on linkage disequilibrium (LD) or a mutation scan of* GRM3*, a replication study using a larger sample and based on LD may be required for conclusive results.

## 1 INTRODUCTION

The glutamate hypothesis is one of the prevailing hypotheses for the pathophysiology of schizophrenia [1]. Also, a recent clinical study showed that LY379268, an agonist of metabotropic glutamate 2/3 receptor (mGluR2/3) that is involved in group II mGluR regulate glutamate neurotransmission through a presynaptic negative regulatory mechanism, has an effect on psychotic symptoms in schizophrenia almost equivalent to that of olanzapine [1].

Several investigations have suggested that metabotropic glutamate 3 receptor (mGluR3) gene has an association with schizophrenia [2-4]. Since the symptoms of methamphetamine-induced psychosis are similar to those of paranoid type schizophrenia [5], it would seem that the mGluR3 gene (*GRM3*) is a good candidate gene for the pathogenesis of methamphetamine-induced psychosis. To evaluate the association between *GRM3* and methamphetamine-induced psychosis, we conducted a case-control study of Japanese samples (181 methamphetamine-induced psychosis patients and 232 controls). The mGluR3 gene (*GRM3* OMIM *601115, 6 exons in a genomic region spanning 221.763Kb) is at 7q21. We selected rs6465084 in *GRM3*, which is reported to be associated with prefrontal brain functioning. Rs6465084 has been found to be associated with decreased verbal list learning and verbal fluency [3]. In addition, Egan and colleagues reported that the rs6465084 A allele predicted decreased levels of *N*-acetylaspartate in the prefrontal cortex in an *in vivo *study, and suggested that the rs6465084 A allele reduced tissue glutamate levels and synaptic abundance [3].

## 2 MATERIALS AND METHODS

### 2.1 Subjects

The subjects in the association analysis were 181 methamphetamine- induced psychosis patients(155 males and 26females; mean age33.3 ± 11.4) and 232 controls (187males and 45females; mean age36.4 ± 11.3). All subjects were unrelated to each other, ethnically Japanese, and lived in the central area of Japan. The patients were diagnosed according to DSM-IV criteria with consensus of at least two experienced psychiatrists on the basis of unstructured interviews and a review of medical records. One hundred thirty-seven subjects with METH- induced psychosis also had dependence on drugs other than METH. Subjects with METH-induced psychosis were excluded if they had a clinical diagnosis of psychotic disorder, mood disorder, anxiety disorder or eating disorder. More detailed characterizations of these subjects have been published elsewhere [6-8]. All healthy controls were also psychiatrically screened based on unstructured interviews including current and past psychiatric history. None had severe medical complications such as cirrhosis, renal failure, heart failure or other Axis-I disorders according to DSM-IV. No structured methods were used to assess psychiatric symptoms in the controls, which included hospital staff, their families and medical students.

The study was described to subjects and written informed consent was obtained from each. This study was approved by the Ethics Committee at Fujita Health University, Nagoya University School of Medicine and and each participating member of the Institute of the Japanese Genetics Initiative for Drug Abuse (JGIDA).

### 2.2 SNP Selection

We selected rs6465084 in *GRM3*, which is reported to be associated with prefrontal brain functioning, for the following association analysis.

### 2.3 SNP Genotyping

SNP genotyping was done using TaqMan assays (Applied Biosystems) [9]. 

### 2.4 Statistical Analysis

Genotype deviation from the Hardy-Weinberg equilibrium (HWE) was evaluated with the chi-square test (SAS/Genetics, release 8.2, SAS Japan Inc, Tokyo, Japan).

Marker-trait association analysis was used to evaluate allele- and genotype-wise association with the chi-square test (SAS/Genetics, release 8.2, SAS Japan Inc, Tokyo, Japan). To control inflation of the type I error rate, we used Bonferroni’s correction. Power calculation was performed using a statistical program prepared by Purcell *et al.*

The significant level for all statistical tests was 0.05.

## 3 RESULTS

Rs6465084 is in HWE. We detected no association between rs6465084 in *GRM3* and METH-induced psychosis in the allele/genotype-wise analysis (Table **1**).

## 4 DISCUSSION

We performed an association study of rs6465084 in *GRM3* and METH- induced psychosis in the Japanese population. Although, we recently found an association between *GRM2* and METH- induced psychosis in the Japanese population [10], in the present study we detected no association between *GRM3* with METH-induced psychosis.

Egan and colleagues reported that the rs6465084 A allele predicted decreased levels of *N*-acetylaspartate in the prefrontal cortex in an *in vivo *study, and suggested that the rs6465084 A allele reduced tissue glutamate levels and synaptic abundance [3]. This influence of *GRM3 *on prefrontal cortex and cognitive function suggests that abnormalities in glutamate neurotransmission may be involved in the pathophysiology of METH- induced psychosis. However, we did not detect an association between *GRM3* and METH-induced psychosis. We designed the present study based on common disease–common variants hypothesis (CD–CV hypothesis) [11]. In addition, we selected only one SNP in *GRM3*. A recent study has showed an association between common diseases such as schizophrenia and rare variants. If the genetic background of METH-induced psychosis is accurately described by the common disease-rare variants hypothesis, further investigation such as medical resequencing using larger samples will be required [8, 12-17].

There are a few limitations in this study. First, the lack of association may be due to biased samples, such as small sample sizes. Second, we selected only one SNP in *GRM3*. To overcome these limitations, it will be necessary conduct a replication study using gene based association analysis and larger samples.

## 5 CONCLUSION

Our results suggest that rs6465084 in *GRM3* does might not play a role in the pathophysiology of METH-induced psychosis in the Japanese population. However, our results have limitations and replication study using gene-based association analysis and larger samples will be required.

## Figures and Tables

**Table 1:** Association Analysis of *GRM3* with MAP with Psychosis

SNP	Phenotype^a^	MAF^b^	N	Genotype Distribution			*P*-Value^c^		
				TT	TC	CC	HWE	Genotype	Allele
rs6465084	Controls	0.0926	232	193	35	4	0.117	0.153	0.941
A>G	MAP with psychosis	0.0911	181	148	33	0	0.177		

a MAP with psychosis: methanphetamine use disorder with psychosis

b MAF: major allele frequency

c Hardy-Weinberg equilibrium.
